# Kinematic Parameters for Tracking Patient Progress during Upper Limb Robot-Assisted Rehabilitation: An Observational Study on Subacute Stroke Subjects

**DOI:** 10.1155/2019/4251089

**Published:** 2019-10-21

**Authors:** Michela Goffredo, Stefano Mazzoleni, Annalisa Gison, Francesco Infarinato, Sanaz Pournajaf, Daniele Galafate, Maurizio Agosti, Federico Posteraro, Marco Franceschini

**Affiliations:** ^1^Department of Neurorehabilitation, IRCCS San Raffaele Pisana, Rome, Italy; ^2^The BioRobotics Institute, Scuola Superiore Sant'Anna, Pisa, Italy; ^3^Rehabilitation Bioengineering Laboratory, Volterra, Italy; ^4^Rehabilitation Medicine Service, NHS-University Hospital of Parma, Parma, Italy; ^5^Rehabilitation Department, Versilia Hospital, AUSL Tuscany North West, Camaiore, Italy; ^6^San Raffaele University, Rome, Italy

## Abstract

**Background:**

Upper limb robot-assisted therapy (RT) provides intensive, repetitive, and task-specific treatment, and its efficacy for stroke survivors is well established in literature. Biomechanical data from robotic devices has been widely employed for patient's assessment, but rarely it has been analysed for tracking patient progress during RT. The goal of this retrospective study is to analyse built-in kinematic data registered by a planar end-effector robot for assessing the time course of motor recovery and patient's workspace exploration skills. A comparison of subjects having mild and severe motor impairment has been also conducted. For that purpose, kinematic data recorded by a planar end-effector robot have been processed for investigating how motor performance in executing point-to-point trajectories with different directions changes during RT.

**Methods:**

Observational retrospective study of 68 subacute stroke patients who conducted 20 daily sessions of upper limb RT with the InMotion 2.0 (Bionik Laboratories, USA): planar point-to-point reaching tasks with an “assist as needed” strategy. The following kinematic parameters (KPs) were computed for each subject and for each point-to-point trajectory executed during RT: movement accuracy, movement speed, number of peak speed, and task completion time. The Wilcoxon signed-rank tests were used with clinical outcomes. the Friedman test and post hoc Conover's test (Bonferroni's correction) were applied to KPs. A secondary data analysis has been conducted by comparing patients having different severities of motor impairment. The level of significance was set at *p* value < 0.05.

**Results:**

At the RT onset, the movements were less accurate and smoothed, and showed higher times of execution than those executed at the end of treatment. The analysis of the time course of KPs highlighted that RT seems to improve the motor function mainly in the first sessions of treatment: most KPs show significant intersession differences during the first 5/10 sessions. Afterwards, no further significant variations occurred. The ability to perform movements away from the body and from the hemiparetic side remains more challenging. The results obtained from the data stratification show significant differences between subjects with mild and severe motor impairment.

**Conclusion:**

Significant improvements in motor performance were registered during the time course of upper limb RT in subacute stroke patients. The outcomes depend on movement direction and motor impairment and pave the way to optimize healthcare resources and to design patient-tailored rehabilitative protocols.

## 1. Introduction

The recovery of upper limb motor impairment after stroke requires prolonged periods of rehabilitation treatment, even if started at an early stage, and the prognosis for functional recovery is often worse than that of lower extremities. The functional recovery process requires a complex integration of muscle activities involving proximal and distal regions of the upper limb, and the execution of movements away from the body and from the hemiparetic side is usually rather challenging [1]. Published studies suggest that there is a highly predictable poor outcome for the return of isolated arm or hand movements 6 months after stroke on the basis of the Fugl-Meyer motor scores [2, 3]. Furthermore, upper limb functional impairment occurs in up to 85% of stroke survivors with a significant long-term impact on activities of daily living (ADLs) and quality of life [4]. Since stroke rehabilitation is often described as a process of active motor relearning, motor task repetition and intensity of the treatment can play an important role in rehabilitation, since they promote neuroplasticity and improve the functional outcome [5, 6].

Robot-assisted therapy (RT) is able to provide high-intensive, repetitive, task-specific, and interactive treatment of the impaired upper limb. In addition, RT is a safe, reproducible, and customizable rehabilitation treatment for promoting the motor learning [7–9]. The efficacy of poststroke RT in improving motor and functional outcomes, and the acceptability are well established in literature [10–13].

Clinical studies on RT usually assess patient's motor ability with a traditional approach based on ordinal measurement scales, which are administered to patients at the beginning and at the end of the period of treatment [11]. However, robotic devices not only provide an assisted upper limb mobilisation but also include sensors that gather biomechanical data during the therapy with a high level of resolution and accuracy [14, 15]. Therefore, robots for rehabilitation provide objective built-in data that can be used to derive measures related to subject's motor impairment. Such movement-related measures allow to quantitatively and ecologically track patient progress over a period of time, providing clinicians greater insight into how components of motor control and coordination change day-by-day with recovery [16–18].

In poststroke upper limb RT, built-in measures have been widely employed for a quantitative patient's assessment [19–38], and they have been classified into kinematic parameters, kinetic parameters, and neuromechanical parameters [16]. A recent review by Tran et al. [39] associated the kinematic parameters (KPs) to the International Classification of Functioning, Disability and Health (ICF) domains. The KPs have been moderately correlated to clinical outcome measures [15, 20, 24] and their validity and reliability have been established [40–42]. However, the majority of published studies employed KPs for assessing patient's status only at the beginning and at the end of the period of treatment [19–24, 26, 35–37]. However, robots for rehabilitation register data during each session of RT and thus allow day-to-day tracking of motor performance [16–18]. Some studies proposed mathematical approaches for modelling the temporal evolution of KPs during RT [25, 28–30, 32–34, 38], which the aim of deeper understanding of the functional and physiological mechanisms underlying the time course of recovery. These models are generally based on the analysis of the overall end-effector trajectory, although it was composed by as set of point-to-point movements having different directions in the workplace. To our knowledge, only Panarese et al. [30] analysed the submovements, each in a different direction, finding that motor recovery was direction-dependent. Other published studies are aimed at understanding whether built-in movement measures could be employed in clinical practice for optimizing the length of poststroke RT [27, 31]. To this extent, Mazzoleni et al. [27] analysed KPs registered by 25 subacute stroke subjects during RT with a planar end-effector robot and found that kinematics significantly improved in the first sessions of treatment, and that a plateau occurred after 10^th^ session. These results were confirmed in a subsequent study by the same group on 12 subacute and 12 chronic stroke patients [31]. These outcomes were encouraging although a restricted number of patients was recruited, and the analysis of KPs did not investigate whether the recovery was dependent from the direction of the movement. Thus, additional research with a higher number of stroke patients is needed in order to understand how kinematic data from robotics devices can be exploited in clinical practice for optimizing and personalizing the RT.

The goal of this retrospective study is to analyse built-in kinematic data registered by a planar end-effector robot for assessing the time course of motor recovery and patient's workspace exploration skills. For that purpose, kinematic data recorded by a planar end-effector robot have been processed for investigating how motor performance in executing point-to-point trajectories with different directions changes during RT. A comparison of subjects having mild and severe motor impairment has been also conducted. The results of this study could help the clinicians to optimise poststroke upper limb RT in terms of length of the therapy and direction of point-to-point movements that need a more intensive training.

## 2. Materials and Methods

### 2.1. Subjects and Clinical Assessment

An observational retrospective study was conducted on a database of 271 inpatients who underwent upper limb RT with the InMotion 2.0 robot (Bionik Laboratories, Watertown, MA, USA) at the IRCCS San Raffaele Pisana of Rome between January 2011 and December 2017.

Inclusion criteria for the patient selection were age between 18 and 80 years, first event of unilateral hemiparetic stroke, subacute phase (RT started within 30 ± 7 days poststroke), upper limb Chedoke-McMaster scores between 2 and 5, and RT for 20 sessions.

Exclusion criteria were bilateral impairment, chronic phase, RT for less than 20 sessions, RT interruption for more than 3 consecutive days, presence of other severe medical conditions, and incomplete data in the database.

The following demographic data have been extracted from the electronic medical records: age, sex, aetiology, stroke location, and distance from the acute event.

The following clinical assessments were registered at the beginning (T1) and at the end (T2) of the period of treatment: modified Barthel Index (BI), which is a measure of ADLs and depicts the degree of independence of a patient from any assistance; Motricity Index of the impaired upper limb (MIul), which assesses the arm motor impairment and ranges from 0 to 100 [43]. These clinical outcome measures are usually delivered as routine clinical assessments. Patient's privacy was preserved by identifying each record in the database by means of a unique alphanumeric code.

### 2.2. Ethical Considerations

Since March 2012, the Italian Data Protection Authority (Garante per la protezione dei dati personali) declared that IRCCS (Istituto di Ricovero e Cura a Carattere Scientifico—Institute for Scientific Research and Health Care) can perform retrospectives studies without the approval of the local Ethical Committee [44] since only a formal communication is needed. Such communication has been registered by the Ethical Committee of the IRCCS San Raffaele Pisana of Rome (date: 22/02/2017; code number: 06/17) that waived the need of participants' consent.

### 2.3. Robot-Assisted Therapy

All subjects conducted 20 daily sessions of upper limb RT by using the InMotion2 system (Bionik Laboratories, Watertown, MA, USA), which is a two-DOF robotic device designed for neurological applications. The subject's arm was placed in a support attached to the robot end-effector and performed eight-direction planar point-to-point reaching task with an “assist as needed” strategy. We followed the methods of Franceschini et al. 2018 [45].

**Figure 1:**
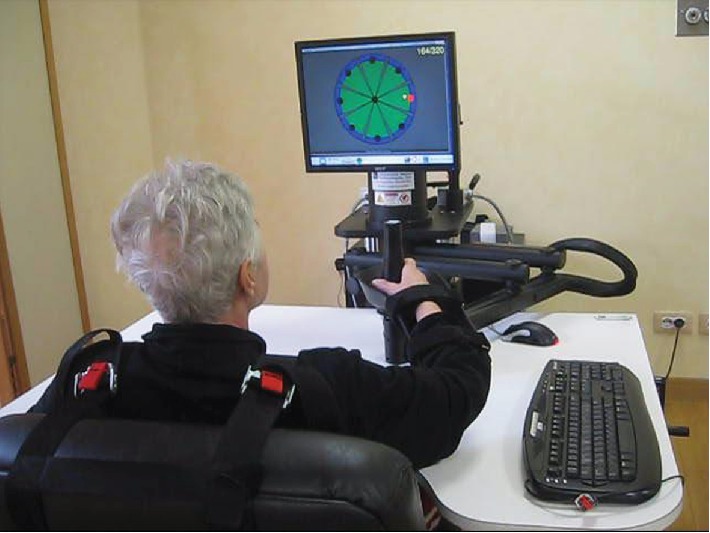
Upper limb RT based on the InMotion 2.0 robotic system.

Each task involved the training of different muscle synergies, moving the end-effector from a central target to 8 peripheral targets, equally spaced on a 0.14 m radius circumference and vice versa (Figure 1). A visual biofeedback was delivered from a monitor placed in front of the subject. The duration of each session was fixed to 45 minutes, as in published clinical studies on poststroke upper limb RT with the same device [12, 20, 35, 45]. The number of repetitions of each session was planned as follows: (i) a series of 16 assisted clockwise repetitions to each target (training), (ii) a series of 16 unassisted clockwise repetitions to each target (record 1), (iii) 3 series of 320 assisted clockwise repetitions (adaptive), and (iv) a series of 16 unassisted clockwise repetitions to each target (record 2). However, since the number of repetitions in the record 1 and record 2 series depended on the patient's residual upper limb abilities, not all patients were able to execute all planned unassisted repetitions.

Every missed session was retrieved and subjects who were not able to retrieve sessions, or interrupted the treatment for more than 3 consecutive days, were excluded from the study.

In addition, all patients underwent conventional physiotherapy sessions according to the standardised rehabilitation protocol for subacute stroke patients of IRCCS San Raffaele Pisana in Rome. The following treatments were provided by senior physical therapists: assisted stretching, shoulder and arm exercises, and functional reaching tasks.

### 2.4. Kinematic Parameters

**Figure 2:**
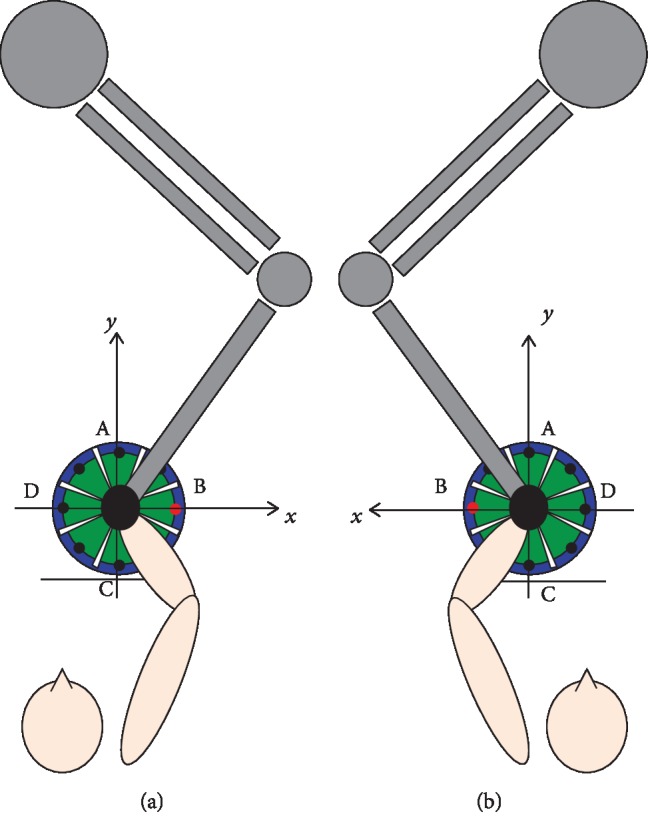
Experimental setup and reference system in case of left (a) or right (b) affected limb.

Kinematic data were recorded at the end-effector robot during the record 1 and record 2 series at a sampling frequency of 200 Hz. As subjects used the robot with the hemiparetic upper limb, the position of the end-effector over time has been expressed with respect to a reference system consistent with the lesion side (Figure 2).

We processed the data of the second unassisted clockwise repetition of the record 2 series, with a customised MATLAB® routine. Then, we downsampled the data, considering the 1^st^, 5^th^, 10^th^, 15^th^, and 20^th^ sessions of RT and we calculated the following KPs for each trajectory from the central target to the peripheral ones: movement accuracy (MovAc), movement speed (MS), number of peak speed (nPS), and task completion time (TCT). These KPs described functional abilities [27, 29] and are representative of two different ICF domains [39]: MovAc, MS, and nPS are in the “body function and structure” domain, while TCT belongs to the “activities” one. The KPs computed in this study are considered as “performance metrics” for assessing the quality of the movement by assuming that normal reaching movements are straight, accurate, smoothed, and fairly quick [17, 18].

The MovAc is a measure of accuracy: the value is 0 if the trajectory lies exactly on a straight line connecting the targets. It is computed as the mean absolute value of the minimum distance of each point of the actual path travelled by the subject from the ideal one (i.e., the straight line connecting the targets).

The MS has been computed from the discrete-time velocity signals *v*_*x*_[*k*] and *v*_*y*_[*k*] along the *x* and *y* axes, respectively (the reference coordinate system is shown in Figure 2), as the mean value of the resultant velocities in the *xy* plane:

(1) $$\begin{array}{c} \text{MS}=\frac{1}{N}\overset{N}{\underset{k=1}{\sum }}\sqrt{{\left(v_{x}\left[k\right]\right)}^{2}+{\left(v_{y}\left[k\right]\right)}^{2}}, \end{array}$$

where *N* is the number of samples for each trajectory.

The nPS is a metric used for assessing the smoothness of the movement in stroke patients [24]: low nPS values derive from few accelerations and decelerations, i.e., smooth movement. The nPS is defined as the number of peaks of the resultant velocity:

(2) $$\begin{array}{c} v_{xy}\left[k\right]=\sqrt{{\left(v_{x}\left[k\right]\right)}^{2}+{\left(v_{y}\left[k\right]\right)}^{2}}. \end{array}$$

The TCT is the time required to carry out each single point-to-point trajectory from the central target to the peripheral one.

Therefore, the KP values have been calculated for each subject and for each point-to-point trajectory executed at the 1^st^, 5^th^, 10^th^, 15^th^, and 20^th^ sessions of RT.

The time course of motor recovery was studied by considering the point-to-point trajectories from the central target to the four principal targets depicted in Figure 2. Since the reference system is consistent with the lesion side, each target corresponded to specific anatomical joint movements (Table 1). Therefore, the considered point-to-point trajectories described different muscle synergies involved for the execution of the reaching tasks [46, 47].

Patient's workspace exploration skills, i.e., the capacity to execute movements towards all peripheral targets, were described at T1 (1^st^ session) and T2 (20^th^ session): the KPs were averaged, normalised between the minimum (0 value) and the maximum (1 value), and depicted in a polar diagram.

A secondary data analysis has been conducted by stratifying patients with respect to the severity of motor impairment at baseline, assessed with the MIul. A recent study on outcome predictors after upper limb RT with the same robot [45] found that subacute stroke patients whose MIul score was higher than 48 at T1 have higher probability to increase their independence in ADLs at T2. For this reason, patients were divided into two groups: subjects with mild motor impairment (MIul > 48) and those with severe motor impairment (MIul ≤ 48) at T1. The intergroup comparison has been conducted for each point-to-point trajectory executed at the 1^st^, 5^th^, 10^th^, 15^th^, and 20^th^ sessions of RT.

### 2.5. Statistics

Descriptive statistics were computed in order to appropriately explain the characteristics of the sample. Data are represented as frequency (with the relative percentage), mean value with standard deviation (SD), and median value with interquartile range (IQR) for the categorical, continuous, and ordinal variables, respectively.

Wilcoxon signed-rank tests were used to find significant differences in ordinal clinical variables. To detect intrasubject differences of the KPs during the time course of the rehabilitation period, a nonparametric repetitive-dependent measure test was applied (Friedman test). Conover's test was used for the post hoc analysis to locate significant differences between sessions. Bonferroni's correction was applied for multiple comparisons. The Mann-Whitney *U* tests were used to compare the KPs (for each movement direction and RT session) of subjects with mild motor impairment (MIul > 48 at T1) with the ones obtained from subjects with severe impairment (MIul ≤ 48 at T1).

For all statistical analyses, the *α* value was set at *p* value < 0.05 and the software was SPSS, version 20.0 (SPSS Inc., Chicago, IL, USA, 2004).

## 3. Results

**Figure 3:**
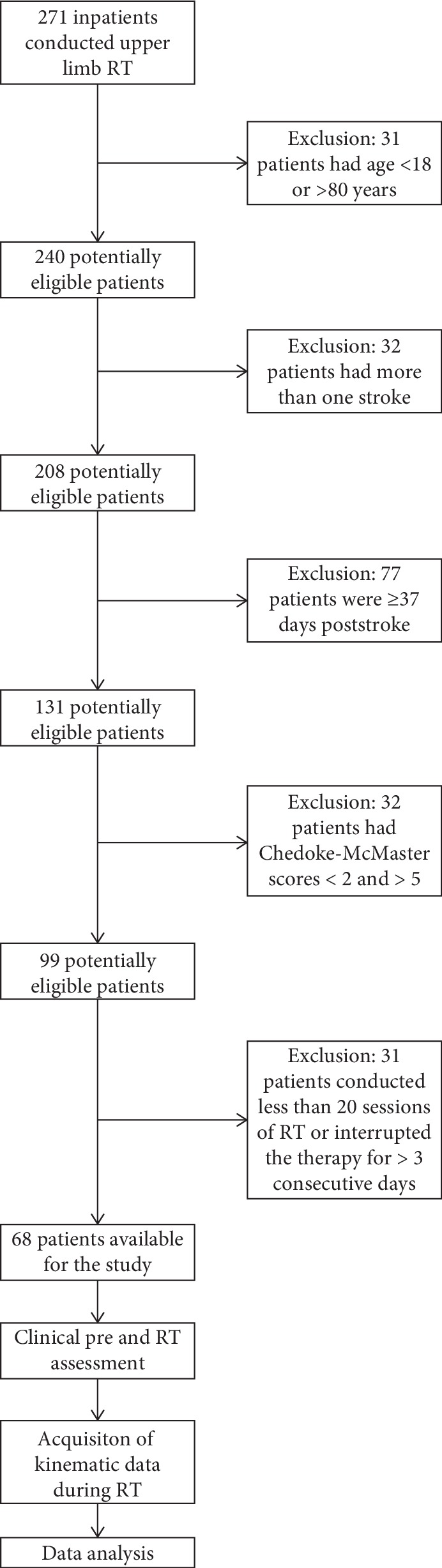
Consort diagram.

Starting from the 271 patients, 68 hemiparetic ischemic and haemorrhagic stroke subjects satisfied the inclusion criteria and were recruited in the study (Figure 3). The mean age was 65.28 years (SD 12.71 years), 23 (33.82%) patients were female, and 21 (30.88%) subjects were affected by stroke on the right side.  Table 2 shows the demographic characteristics of the sample at baseline and the clinical scores (BI and MIul) at T1 and T2. At the end of RT, the clinical outcomes show an increase in ADLs and in motor function of the paretic upper limb: the Wilcoxon signed-rank tests evidenced statistically significant improvements in both BI (*p* value < 0.001) and MIul (*p* value < 0.001) scores, in accordance with studies on the efficacy of RT in stroke survivors [11, 12].

The nonparametric repetitive-dependent measure tests (Friedman tests) were applied to each point-to-point trajectory and for each KP. The analysis did not reveal a significant difference for the overall MovAc changes in all movement directions. Conversely, significant temporal differences were found in MS in the movements towards the targets A (*χ*^2^ = 22.04; *p* value < 0.001), B (*χ*^2^ = 33.96; *p* value < 0.001), C (*χ*^2^ = 44.84; *p* value < 0.001), and D (*χ*^2^ = 34.44; *p* value < 0.001). An analogous result was obtained on nPS: targets A (*χ*^2^ = 53.83; *p* value < 0.001), B (*χ*^2^ = 33.27; *p* value < 0.001), C (*χ*^2^ = 31.49; *p* value < 0.001), and D (*χ*^2^ = 39.18; *p* value < 0.001). Similarly, the overall temporal decrease of TCT was significant in all movement directions: targets A (*χ*^2^ = 59.98; *p* value < 0.001), B (*χ*^2^ = 44.08; *p* value < 0.001), C (*χ*^2^ = 42.80; *p* value < 0.001), and D (*χ*^2^ = 45.83; *p* value < 0.001).

**Figure 4:**
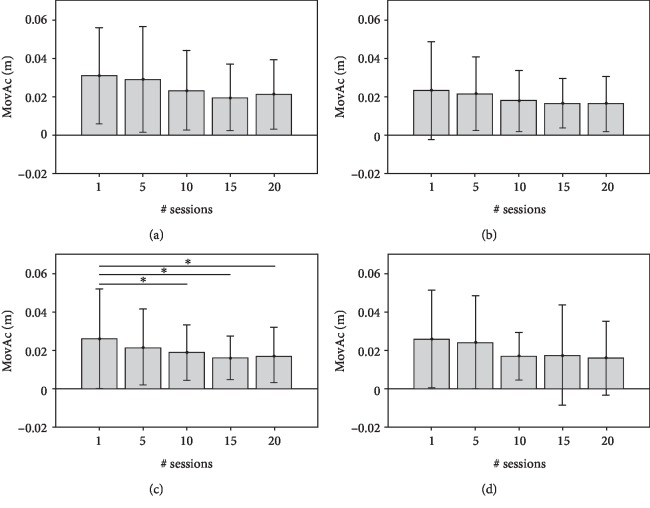
MovAc values (mean and standard deviation) at 1^st^, 5^th^, 10^th^, 15^th^, and 20^th^ sessions of RT and significant post hoc comparisons between sessions (Conover's test): ^∗^*p* value < 0.05; ^∗∗^*p* value < 0.001; ^∗∗∗^*p* value < 0.0001. The data obtained by analysing the end-effector trajectories towards the four targets (A, B, C, D) are showed separately.

**Figure 5:**
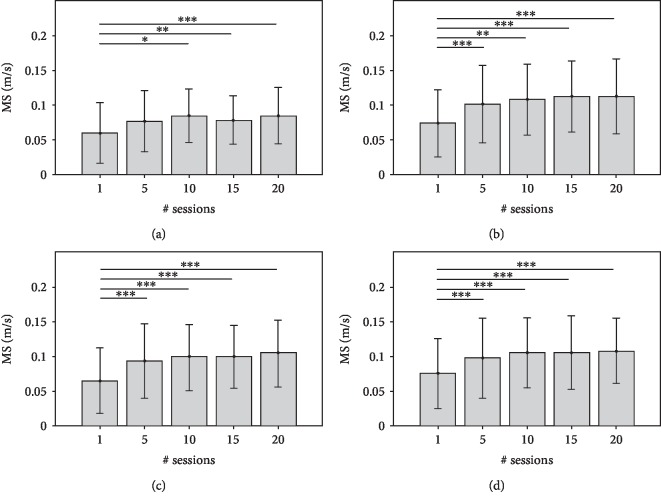
MS values (mean and standard deviation) at 1^st^, 5^th^, 10^th^, 15^th^, and 20^th^ sessions of RT and significant post hoc comparisons between sessions (Conover's test): ^∗^*p* value < 0.05; ^∗∗^*p* value < 0.001; ^∗∗∗^*p* value < 0.0001. The data obtained by analysing the end-effector trajectories towards the four targets (A, B, C, D) are showed separately.

**Figure 6:**
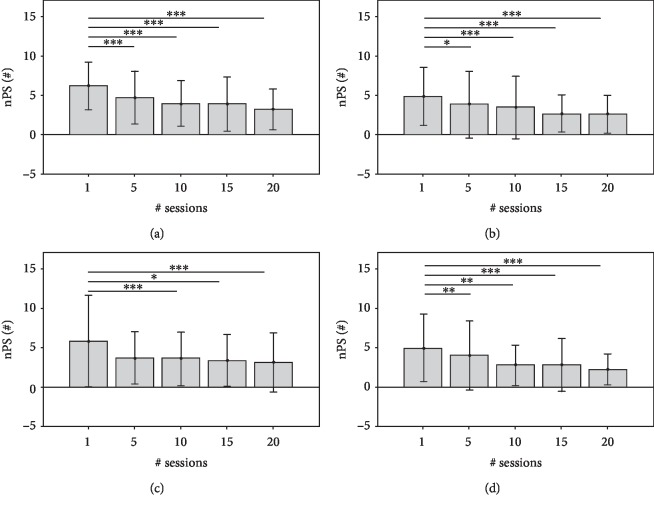
nPS values (mean and standard deviation) at 1^st^, 5^th^, 10^th^, 15^th^, and 20^th^ sessions of RT and significant post hoc comparisons between sessions (Conover's test): ^∗^*p* value < 0.05; ^∗∗^*p* value < 0.001; ^∗∗∗^*p* value < 0.0001. The data obtained by analysing the end-effector trajectories towards the four targets (A, B, C, D) are showed separately.

**Figure 7:**
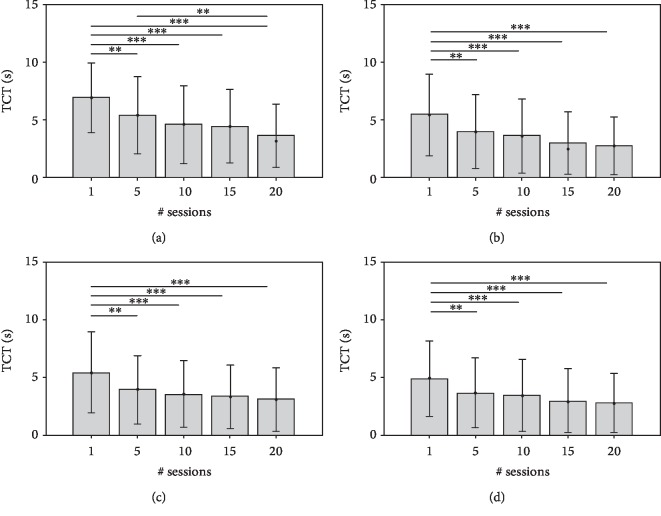
TCT values (mean and standard deviation) at 1^st^, 5^th^, 10^th^, 15^th^, and 20^th^ sessions of RT and significant post hoc comparisons between sessions (Conover's test): ^∗^*p* value < 0.05; ^∗∗^*p* value < 0.001; ^∗∗∗^*p* value < 0.0001. The data obtained by analysing the end-effector trajectories towards the four targets (A, B, C, D) are showed separately.

The Figures 4–7 show each KP (mean values and SDs) obtained from the point-to-point trajectories (A, B, C, D) executed by the 68 subjects at the 1^st^, 5^th^, 10^th^, 15^th^, and 20^th^ sessions of RT. The statistical analysis of intersession difference is shown with a representation of the corresponding *p* values, obtained with the post hoc Conover's tests (Bonferroni correction). At the RT onset (1^st^ session), all point-to-point movements are characterised by curved trajectories (mean MovAc at T1 = 0.019 m) with distinct sub movements (mean nPS at T1 = 4.47) executed at low mean speed (mean MS at T1 = 0.064 m/s) and with a high time of execution (mean TCT at T1 = 5.69 s). At the end of treatment, the data are significantly different: mean MovAc at T2 = 0.017 m, mean nPS at T2 = 2.81, mean MS at T2 = 0.10 m/s, and mean TCT at T2 = 0.10 s.

The MovAc (Figure 4) represents the accuracy (low values represent straighter movements) of the trajectory, and it decreases during the course of the treatment. Such behaviour is noticeable in all movement directions, with a significant trend in the tasks towards the target C. The tasks that involve the elbow extension and shoulder internal rotation movements (i.e., reaching the target A) are characterised by higher MovAc values both at the 1^st^ (mean MovAc = 0.03 m) and at the 20^th^ sessions (mean MovAc = 0.018 m). The trajectories towards target C have a significant decrease of MovAc after the 10^th^ session, and the values are sustained afterwards. The mean MovAv obtained from the movement direction A were 0.03 m at the 1^st^ session, of 0.023 m at the 10^th^ session, and of 0.018 m at the 20^th^ session. Reaching the target C registered a mean value of MovAc of 0.026 m at the 1^st^ session, of 0.019 m at the 10^th^ session, and of 0.017 m at the 20^th^ session. Data from trajectories towards target D were characterised by a mean MovAc of 0.026 m at the 1^st^ session, of 0.017 m at the 10^th^ session, and of 0.016 m at the 20^th^ session. Movements towards the targets A, B, and D do not have significant intersession changes of MovAc.

The MS (Figure 5) increases in all movement directions during RT. The mean speed significantly changed after the 5^th^ session, and the values are maintained in the subsequent ones. Analogous to MovAc, the MS highlights lower performances of the tasks that involve the elbow extension and shoulder internal rotation (i.e., reaching target A) that show the lowest velocities (mean MS of 0.06 m/s, 0.084 m/s, 0.084 m/s at the 1^st^, 10^th^, and 20^th^ sessions, respectively). The remaining tasks almost doubled their movement speed after the 5^th^ session and such value persists over time. For example, the mean MS of target B was 0.073 m/s, 0.010 m/s, 0.011 m/s at the 1^st^, 10^th^, and 20^th^ sessions, respectively. Significant intersession variations were registered between the 1^st^ session and the following ones in all movement directions.

The nPs (Figure 6) represents the smoothness (low values represent high smoothness) of the trajectory which decreases in all the movement directions. Thus, the patients tended to have less distinct submovements during the course of RT. For instance, the mean numbers of peaks in movements toward the target A were 6.23 at the 1^st^ session, 4.00 at the 10^th^ session, and 3.25 at the 20^th^ session of RT. A plateau trend after the 5^th^ session is found in tasks towards targets A, B, and C.

Movements towards target C showed a significant variation of smoothness after the 10^th^ session.

A similar decreasing temporal evolution is found in TCT values, where trajectories towards the target A were characterised by higher times of execution at every session. Specifically, the mean TCT obtained from the movement direction A were 6.93 s at the 1^st^ session, of 4.55 s at the 10^th^ session, and of 3.58 s at the 20^th^ session. In all movement directions, the post hoc intersession analysis revealed significant differences between the 1^st^ session and the following ones.

**Figure 8:**
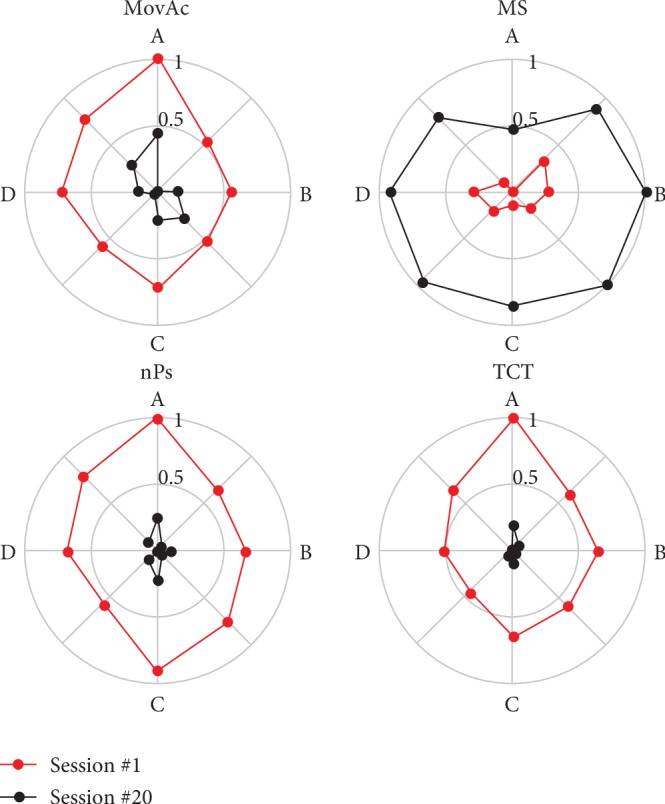
Polar plots of KP changes. Each figure represents the mean values of each KP at the start (red line) and at the end (black line) of RT. The changes of each KP are normalised between the minimum (0 is the circle centre) and the maximum (1 is the circle border) values.

Figure 8 describes patient's workspace exploration skills at T1 (red line) and T2 (black line). Each point of the polar plots represents the mean values of the normalised KP with respect to the 8 movement directions. At T1, the trajectories towards target A showed the highest values on MovAc, nPs, and TCT, and the lowest value of MS. The figures show a motor improvement after RT in the trajectories towards all targets. At T2, the MovAc is still high towards target A (0.25), while in the other directions is about 0.08. MS increased to the maximum value in all trajectories, except to the one towards target A, where the speed at the end of RT is half of the others. The nPS and TCT decrease in all point-to-point trajectories, although the movements towards targets A and C have keep higher values also at T2.

The secondary data analysis divided the sample into 2 groups with a criteria based on the motor impairment at T1: specifically 37 patients had MIul ≤ 48, and 31 patients had MIul > 48.

Patients with severe upper limb motor impairment (MIul ≤ 48) showed higher values of MovAc in all point-to-point trajectories and RT sessions (mean MovAc of 0.022 m at the 1^st^ session, of 0.023 m at the 10^th^ session, and of 0.020 m at the 20^th^ session) than patients with MIul > 48 (mean MovAc of 0.020 m at the 1^st^ session, of 0.015 m at the 10^th^ session, and of 0.011 m at the 20^th^ session). Significant intergroup MovAc differences were registered in movements towards target A (*W* = 813.00; *p* value = 0.003) and target B (*W* = 745.00; *p* value = 0.003) at the 15^th^ session, towards target C at the 20^th^ session (*W* = 734.50; *p* value = 0.005), and towards target D and the 1^st^ (*W* = 776.00; *p* value = 0.013), 10^th^ (*W* = 822.50; *p* value = 0.002), 15^th^ (*W* = 804.00; *p* value = 0.005), and 20^th^ (*W* = 867.50; *p* value < 0.001) sessions.

Subjects with MIul > 48 executed faster trajectories (higher MS values) than their peers with MIul ≤ 48. Significant intergroup differences were found in all sessions for movements towards target A (*W* = 0.00; *p* value < 0.001), in the 1^st^ session for directions B (*W* = 385.00; *p* value = 0.02) and C (*W* = 400.50; *p* value = 0.03), and the 10^th^ session for directions B (*W* = 392.50; *p* value = 0.03) and D (*W* = 395.00; *p* value = 0.03).

The number of peaks of the resultant velocity was always higher in the group with more severe impairment. In subjects with MIul ≤ 48, the mean numbers of peaks of trajectories toward the target A were 6.45 at the 1^st^ session, 2.84 at the 10^th^ session, and 3.59 at the 20^th^ session of RT. In subjects with MIul > 48, the nPs toward the target A were 4.29 at the 1^st^ session, 2.47 at the 10^th^ session, and 2.31 at the 20^th^ session of RT. The Mann-Whitney *U* test revealed significant differences in movements towards target A (1^st^ session: *W* = 784.00, *p* value = 0.01; 5^th^ session: *W* = 754.50, *p* value = 0.02; 15^th^ session: *W* = 842.00, *p* value < 0.001), B (1^st^ session: *W* = 757.00, *p* value = 0.02; 5^th^ session: *W* = 728.00, *p* value = 0.04; 10^th^ session: *W* = 732.50, *p* value = 0.04), C (5^th^ session: *W* = 784.00, *p* value = 0.01; 10^th^ session: *W* = 859.00, *p* value < 0.001; 15^th^ session: *W* = 773.00, *p* value = 0.01), and D (1^st^ session: *W* = 814.50, *p* value = 0.0003).

The TCT values confirmed the trend of other KPs: subjects having severe upper limb impairment had significant higher time of execution than subjects with mild impairment did. The intergroup analysis showed significant differences in movements towards target A (1^st^ session: *W* = 734.50, *p* value = 0.04; 15^th^ session: *W* = 843.50, *p* value < 0.001), B (5^th^ session: *W* = 761.50, *p* value = 0.02; 10^th^ session: *W* = 754.50, *p* value = 0.03; 15^th^ session: *W* = 740.00, *p* value = 0.04), C (1^st^ session: *W* = 758.50, *p* value = 0.02; 5^th^ session: *W* = 750.00, *p* value = 0.03; 10^th^ session: *W* = 835.50, *p* value = 0.001; 15^th^ session: *W* = 779.00, *p* value = 0.01; 20^th^ session: *W* = 744.50, *p* value = 0.04), and D (1^st^ session: *W* = 813.50, *p* value = 0.0003; 10^th^ session: *W* = 779.00, *p* value = 0.01; 15^th^ session: *W* = 792.50, *p* value = 0.007; 20^th^ session: *W* = 818.00, *p* value =0.0027).

## 4. Discussion

Kinematic data recorded by a planar end-effector robot during the RT of 68 subacute stroke patients was processed for assessing the time course of motor recovery and patient's workspace exploration skills. A set of KPs, which are representative of motor performance, were calculated, and their changes with respect to time and movement direction were analysed.

The data analysis showed that RT leads to significant improvements in kinematic components of upper motor performance. Changes of movement kinematics have been described in terms of accuracy, velocity, smoothness, and time of execution of the motor tasks.

At the RT onset, the point-to-point trajectories were less accurate and smoothed, and showed higher times of execution than those executed at the end of treatment. These findings are in agreement with studies [25, 27, 29, 31] that associated the variations of KPs to motor recovery, registering an improvement of KPs during the period of treatment.

The analysis of the time course of KPs highlighted that RT seems to improve the motor function mainly in the first sessions of treatment: most KPs showed significant intersession differences during the first 5/10 sessions. Afterwards, no further significant variations occurred. Similar results have been found in studies on a limited number of stroke patients [27, 31].

The descriptive analysis of different movement directions showed that the ability to perform movements away from the body (target A) and from the hemiparetic side (target B) was initially limited: these movements had low accuracy, speed, smoothness, and higher execution times compared with movements toward the body (target C) and toward the hemiparetic side (target D). At the end of the treatment, the workspace was successfully restored, although the movements that involved elbow extensions and shoulder internal rotation (target A) remained rather challenging to be performed. The results are in accordance with similar studies on motor recovery of stroke patients [1, 46] and RT [7, 11, 30] and suggest that different mechanisms are responsible for recovering movements toward different target positions, in agreement with studies on motor synergies in stroke survivors [30, 47].

The results obtained from data stratification evidenced that at T1, the majority of KPs were significantly different (MovAc, target D; MS, targets A, B, C; nPs, targets A, B, D; TCT, targets A, C, D). During the time course of RT, such differences persisted only in trajectories towards target A (MS, nPs), C (TCT), and D (MovAc, TCT). The two groups of patients did not register any significant difference in the other KPs over time.

This study presented several limitations that deserve to be discussed. Firstly, normative reference values of KPs are not available for both healthy subjects and stroke patients who performed conventional upper limb therapy. Secondly, the study employed a planar end-effector robot, while 3D exoskeleton devices for upper limb RT are commercially available. Thirdly, the MovAc values could be influenced by the number of samples of the trajectory and by the systematically curved behaviour of normal reaching movements [48]: it could justify the differences, in terms of statistical outcomes, between MovAc and the other KPs. Finally, the study is retrospective; therefore, it did not assess clinical and kinematic effects of prolonged RT (>20 sessions) and did not include a follow-up assessment. However, since a recent study on the long-term clinical effects (after 6 months) of upper limb RT in subacute stroke patients found that the clinical improvements observed at the end of treatment persisted over time [13], we are confident that such trend could be noticed in KPs too.

The research agenda should include the gathering of normative reference values, the implementation of advanced algorithms for the analysis of movement during RT, and the investigation of recently released devices for 3D upper limb rehabilitation.

## 5. Conclusions

Robotic systems for stroke rehabilitation may be considered as a tool with a twofold aim: (i) training the patient with an assist-as-needed approach and (ii) assisting the clinicians to plan and personalise the rehabilitation treatments. The results obtained by analysing kinematic data from 68 subacute stroke patients showed significant improvements in motor performance in the first 5-10 sessions of RT. Moreover, the recovery was different for each movement direction. Such outcomes are in accordance with literature on the topic [27, 31, 34].

Future studies on a larger sample of subjects may highlight the clinical characteristics of patients who may benefit upper limb RT. Moreover, a more detailed analysis of KPs calculated in the first session of RT may contribute to optimize healthcare resources and to design patient-tailored rehabilitative protocols with an ecological approach.

## Figures and Tables

**Table 1:** Correspondence between the target position and the joint movements.

Target label	Target coordinates (m)	Elbow movement	Shoulder movement
A	(0.00, 0.14)	Extension	Internal rotation and flexion
B	(0.14, 0.00)	Flexion	Abduction
C	(0.00, -0.14)	Flexion	External rotation and extension
D	(-0.14, 0.00)	Extension	Adduction

Target coordinates are expressed as (*x*, *y*), taking into account the reference system shown in Figure 2.

**Table 2:** Characteristics of the sample and clinical outcomes.

Variables	*n* (%)	T1 median (IQR)	T2 median (IQR)	*p* value
Gender, male/female	45 (66.18)/23 (33.82)			
Aetiology, ischemic/haemorrhagic	49 (72.05)/19 (27.95)			
Lesion side, left/right	29 (42.62)/39 (57.35)			
BI		26.50 (9.90-49.00)	79.50 (39.90-97.10)	<0.001
MIul		43.00 (1.00-78.15)	77.00 (14.30-100.00)	<0.001

IQR: interquartile range; BI: modified Barthel Index; MIul: Motricity Index paretic upper limb.

## Data Availability

The authors are available to send data to those who request it.

## Additional Points

*Consent for Publication*. All authors gave their consent for the manuscript publication.
